# Coinfection of COVID-19 and Dengue: A Case Report

**DOI:** 10.3389/fmed.2022.872627

**Published:** 2022-07-27

**Authors:** Chowdhury Nusaiba Binte Sayed Prapty, Nafisa Ahmed, Yusha Araf, Zhijun Yang, Jingbo Zhai, Mohammad Jakir Hosen, Chunfu Zheng

**Affiliations:** ^1^Department of Immunology, School of Basic Medical Sciences, Fujian Medical University, Fuzhou, China; ^2^Biotechnology Program, Department of Mathematics and Natural Sciences, BRAC University, Dhaka, Bangladesh; ^3^Department of Genetic Engineering and Biotechnology, School of Life Sciences, Shahjalal University of Science and Technology, Sylhet, Bangladesh; ^4^Shanghai Omics Biotechnology Co., Ltd., Shanghai, China; ^5^Medical College, Inner Mongolia Minzu University, Tongliao, China; ^6^Key Laboratory of Zoonose Prevention and Control at Universities of Inner Mongolia Autonomous Region, Tongliao, China; ^7^Department of Microbiology, Immunology and Infectious Diseases, University of Calgary, Calgary, AB, Canada

**Keywords:** dengue, COVID-19, coinfection, case, SARS-CoV-2

## Abstract

While the COVID-19 pandemic takes the world by storm, dengue-endemic regions risk developing a co-epidemic in COVID-19/dengue coinfection. With both infections as causes of high morbidity rates, the potentially fatal outcomes of coinfection are even greater, and several cases are emerging, severe and moderate, showing how common it may become in certain regions. The case reported here shows a 38-year-old male patient with high-grade fever, with complaints of nausea, joint, and muscle aches, all characteristic symptoms of COVID-19 and dengue. Initially suspected of being infected with COVID-19 only, the RT-PCR test of the nasopharyngeal swab confirmed COVID-19 infection, while the positive reactivity to IgG and IgM in the Dengue Duo test revealed a dengue coinfection. Except for the persistent high fever, the Patient's symptoms were not severe, although the tests confirmed the infections to be “moderate to severe” and showed steady and rapid recovery. The tests showed some interesting results, which provided additional research opportunities. Overall, this case report illustrates the existence of coinfections in the Philippines, demonstrating the difficulty in distinguishing the two infections and the need for proper diagnosis, prevention, and management measures.

## Introduction

First identified in Wuhan, Hubei, China, in December 2019, the novel coronavirus disease (COVID-19) emerged as an unprecedented outbreak of pneumonia and related respiratory illnesses, which quickly developed into a global pandemic within a few weeks, prompting the World Health Organization (WHO) to declare it as a global emergency (1). This infection is caused by a beta coronavirus called Severe Acute Respiratory Syndrome Coronavirus 2 (SARS-CoV-2). This infection typically affects the lower respiratory tract and causes pneumonia in humans. The virus is fundamentally transmitted through air droplets while coughing or sneezing from person to person (2). As of February 2022, COVID-19 has claimed the lives of more than 5.7 million people worldwide, infecting 396 million (3).

On the other hand, Dengue is the most common arboviral disease globally, particularly affecting the tropical and subtropical regions where the vectors (Aedes mosquitos) are most common, rendering dengue an endemic disease for those regions (4). As the leading cause of hospitalization and casualty by the arthropod-borne viral disease globally, dengue incidences have increased by 30-folds in the last 50 years worldwide (5). Dengue fever can be caused by four distinct serotypes of the dengue virus (DENV 1-4) that belong to the Flaviviridae family and present with symptoms such as high fever, nausea, headaches, myalgia, skin rashes, retro-orbital pains, and arthralgia (6).

Despite distinct pathophysiological differences between the two infections, COVID-19 and dengue share clinical symptoms and laboratory features, making differentiation difficult, especially in dengue-endemic regions, which is a major health concern as it increases the risk of misdiagnosis and creates challenges as the management of the two diseases is completely different (7). For example, in the Philippines, dengue is endemic and placed as one of its eight pervasive infectious diseases, putting the country in the fourth position concerning dengue burden in Asia (8). A coinfection by dengue and SARS-CoV-2 would undoubtedly be a dangerous combination. Several such cases have been reported in the dengue-endemic regions, which can develop into a co-epidemic if not managed cautiously and must be observed and recorded for the physicians and health authorities to make decisions accordingly. This case report presents a 38-year-old male from the Philippines diagnosed with a coinfection.

## Case Presentation

A 38-year-old male living in Laguna, Philippines, was presented with persistent high fever, nausea, arthralgia, and slight complaints of myalgia. No reports of cough, sore throat, or dyspnea were made. The Patient had no prior travel history or contact with COVID-19 patients. He had no record of any chronic illness, had not been vaccinated against COVID-19, and is a strong, well-built adult. After 5 days of continuous fever progressing from 38.7°C to 39.8°C and no effect after taking common over-the-counter fever reducers, the Patient went to the Sta. Rosa Hospital and Medical Center for proper diagnosis.

## Examination

At the initial examination, the Patient's vitals were blood pressure of 110/80 mmHg, pulse rate of 85/min, and respiratory rate of 18/min, all considered within the normal range except the body temperature, which was recorded as 39.4°C. The male did not complain of any upper and lower respiratory tract discomforts or symptoms. The physical examination was unremarkable, with no skin rashes (as expected in dengue). However, the Patient noted frequent mosquito bites in the preceding days, even 2 days before diagnosis. His gums appeared slightly red, although not bleeding, so the physicians opted for a hematology test of complete blood count (CBC), urinalysis, arterial blood gas analysis, general laboratory chemistry report, a CKMB and troponin test for an indication of heart health (Table 1), and SD BIOLINE Dengue Duo as well as a SARS-CoV-2 viral RNA RT-PCR from nasopharyngeal and oropharyngeal swabs since the COVID-19 pandemic was in full swing in the Philippines at the time of this case (August 2021). DENV-1-4 real-time RT-PCR was also performed (Table 2) following the Dengue Duo test.

**Table 1:** Results of all tests performed, including patient's vitals, CBC on three consecutive days, urinalysis, general laboratory chemistry, arterial blood gas analysis, immunology test, and radiography report.

**Tests**	**Result**	**Reference range**	**Interpretation**
**Patient's vitals**			
Blood pressure	110/80 mmHg	<120/80 mmHg	Normal
Pulse rate	85 beats/min	60–100 beats/min	Normal
Respiratory rate	18 breaths/min	12–20 breaths/min	Normal
Body temperature	39.4°C	37°C	High
**CBC test day 1**			
Hemoglobin	182 g/L	130–180 g/L	High
Hematocrit	0.51 g/L	0.42–0.60 g/L	Normal
RBC	5.71 × 10^12^/L	4.50–6.50 × 10^12^/L	Normal
WBC	7.44 × 10^9^/L	4.00–11.00 × 10^12^/L	Normal
MCV	89 fL	78–100 fL	Normal
MCH	31 pg	27–32 pg	Normal
MCHC	358 g/L	310–370 g/L	Normal
RDW	14.5%	11.5–14.0%	High
Platelet	145 × 10^9^/L	150–400 × 10^9^/L	Low
Neutrophils	71%	36–66%	High
Lymphocytes	25%	20–45%	Normal
Monocytes	3%	5–12%	Low
Eosinophils	0%	0–6%	Normal
Basophils	1%	0–2%	Normal
**CBC test day 2**			
Hemoglobin	172 g/L	130–180 g/L	Normal
Hematocrit	0.52 g/L	0.42–0.60 g/L	Normal
RBC	5.70 × 10^12^/L	4.50–6.50 × 10^12^/L	Normal
WBC	7.76 × 10^9^/L	4.00–11.00 × 10^12^/L	Normal
MCV	91 fL	78–100 fL	Normal
MCH	30 pg	27–32 pg	Normal
MCHC	333 g/L	310–370 g/L	Normal
RDW	11.1%	11.5–14.0%	Low
Platelet	112 × 109/L	150–400 × 109/L	Low
Neutrophils	58%	36–66%	Normal
Lymphocytes	32%	20–45%	Normal
Monocytes	10%	5–12%	Normal
Eosinophils	0%	0–6%	Normal
Basophils	0%	0–2%	Normal
**CBC test day 3**			
Hemoglobin	177 g/L	130–180 g/L	Normal
Hematocrit	0.51 g/L	0.42–0.60 g/L	Normal
RBC	5.58 × 10^12^/L	4.50–6.50 × 10^12^/L	Normal
WBC	4.76 × 10^9^/L	4.00–11.00 × 10^12^/L	Normal
MCV	91 fL	78–100 fL	Normal
MCH	31 pg	27–32 pg	Normal
MCHC	348 g/L	310–370 g/L	Normal
RDW	14.6%	11.5–14.0%	High
Platelet	157 × 10^9^/L	150–400 × 10^9^/L	Normal
Neutrophils	65%	36–66%	Normal
Lymphocytes	29%	20–45%	Normal
Monocytes	3%	5–12%	Low
Eosinophils	0%	0–6%	Normal
Basophils	1%	0–2%	Normal
**Urinalysis**			
**Gross:**			
Color	Dark yellow		
Transparency	Hazy		
Reaction	6.0		
Specific gravity	1.030		
**Chemicals:**			
Albumin	2+		
Glucose	Trace		
Leukocytes	3+		
Nitrite	Negative		
Urobilinogen	Normal		
Blood	Trace		
Bilirubin	Negative		
Ketone	Negative		
**Urinalysis microscopic:**			
Red cell	2–4 HPF		
Pus cell	25–30 HPF		
**Urinalysis crystals:**			
Epithelial cells	Moderate		
Bacteria	Heavy		
Mucus threads	Moderate		
**General laboratory chemistry report day 1**			
Blood urea nitrogen	5.82 mM	2.50–6.40 mM	Normal
Creatinine	120 μM	62–115 μM	High
Blood uric acid	383 μM	208–428 μM	Normal
SGPT/ALT	374 U/L	16–63 U/L	Extremely high
Sodium	130 mM	135–148 mM	Low
Potassium	3.9 mM	3.5–5.3 mM	Normal
**General laboratory chemistry report day 2**			
Fasting blood sugar	8.00 mM	4.10–5.90	High
Blood urea nitrogen	5.02 mM	2.50–6.40 mM	Normal
Creatinine	95 μM	62–115 μM	Normal
Total cholesterol	5.65 mM	0–5.20 mM	High
Triglycerides	2.06 mM	0–1.70 mM	High
HDL cholesterol	0.51 mM	0–1.55 mM	Normal
LDL cholesterol	4.2 mM	0–3.3 mM	High
Hemoglobin A1C	8.1%	4.6–6.2%	High
**Arterial blood gas analysis day 1**			
pH	7.441	7.35–7.45	Normal
pCO_2_	29 mmHg	35–45 mmHg	Low
pO_2_	67 mmHg	80–100 mmHg	Low
HCO_3_	19.1 mM	22–26 mM	Low
O_2_ Sat	93%	95–100%	Low
**Arterial blood gas analysis day 2**			
pH	7.406	7.35–7.45	Normal
pCO_2_	27.9 mmHg	35–45 mmHg	Low
pO_2_	99 mmHg	80–100 mmHg	Normal
HCO_3_	20.6 mM	22–26 mM	Low
O_2_ Sat	98%	95–100%	Low
**Immunology test**			
Troponin I	<0.10 ng/mL	0–0.30 ng/mL	Normal
CKMB	2.42 ng/mL	0.00–5.00 ng/mL	Normal
**Radiography report**			
Portable chest X-ray	Ill–defined density, left lower lobe heart is not enlarged The diaphragm and bony thorax are unremarkable		Impression: left lower lobe pneumonia

**Table 2:** Test res for DENV and COVID-19.

**Tests**			**Result**
COVID-19 RT-PCR			Positive
**Dengue panel**			
NS1			Negative
IgM			Positive
IgG			Positive
DENV-1-4 real-time RT-PCR			Positive
SARS-CoV-2 RT-PCR	Viral Load (copies/ml)	Ct value	Positive
	6.78 log10	27	

## Investigation

The Dengue Duo test gave negative results for NS1 antigen but showed positive results for both IgG and IgM anti-dengue antibodies, concluding that the Patient has been affected by dengue in recent weeks. The COVID-19 RT-PCR test came a day later and showed positive for the presence of SARS-CoV-2. However, due to antigenic similarities between DENV and SARS-CoV-2, cross-reactivity between these two viruses can give false-positive results in rapid serology tests for both diseases (2, 9). The positive IgG and IgM test results but negative NS1 results raised the possibility that the Patient had COVID-19, and the SARS-CoV-2 antibodies gave false-positive results in the Dengue Duo Test. Therefore, to rule out this possibility, DENV-1-4 RT-PCR was conducted, which also came positive. Hence, the Patient was confirmed as a co-infected patient. As both IgG and IgM and the RT-PCR test results were positive, it was reasonable to conclude that the NS1 antigen test gave a false negative result. Some ambiguity remained about how the DENV-1-4 RT-PCR showed positive results since DENV nucleic acid can generally be detected only during the early part of the infection while IgM and IgG count increase exponentially with disease progression. A possible reason could be a recurring dengue infection as the Patient complained about continued and frequent mosquito bites.

**Figure 1:**
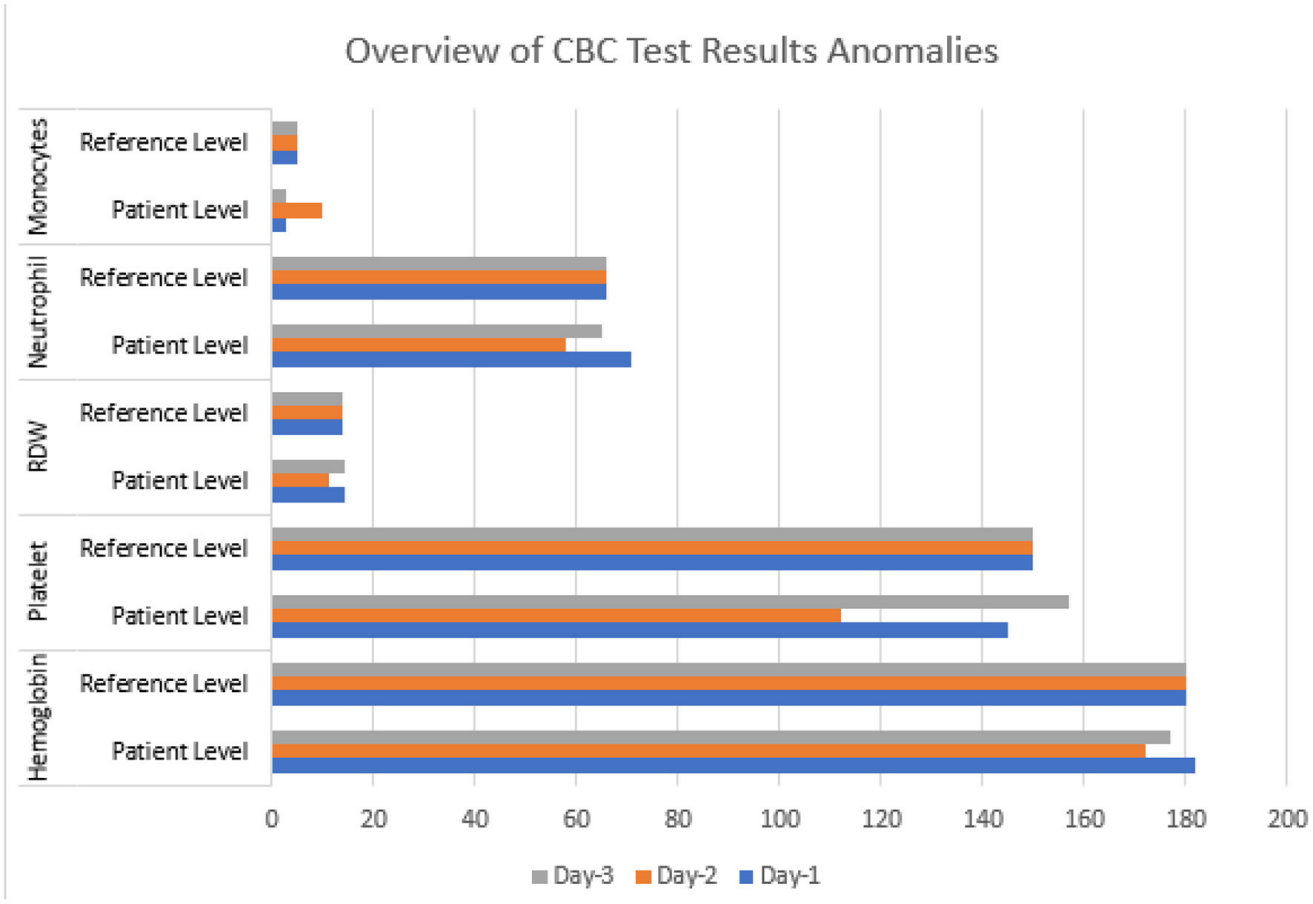
Chart summary of the three CBC test results showing anomalies alongside reference values.

Three hematology tests (CBC) were done on three consecutive days following the disease's progress (Figure 1). On the first test, most components, such as hematocrit, count of lymphocytes, eosinophils, basophils, RBC, WBC, MCV, MCH, MCHC, and RDW, were found to be within the normal range. The neutrophil count was high, and the monocyte count was low. The hemoglobin level was slightly higher at 182 g/L than the acceptable range of 130–180 g/L. However, the major deviation was in the significantly lower levels of platelet count, at 145 ×10^9^/L compared to the normal range of 150–400 ×10^9^/L. On the second test, the hemoglobin had returned to its normal range (172 g/L) and all the remaining normal, except the platelet count, which dropped even more than the previous test, at 112 ×10^9^/L, and this alarmed the attending physicians. The third test was done; however, it showed a rise in platelet count to 157 ×10^9^/L, a value within the normal range, while all the other blood components also showed a healthy range. The variation in the platelet counts on the consecutive results is not conclusive evidence of a coinfection. However, the fluctuations alerted the medical attendants about the possibility of health deterioration resulting in further testing and continued care of the Patient.

The urinalysis on the first day showed normal urine concentration with a healthy color, transparency, pH, and no harmful secretions; only the bacterial count was “heavy,” and the Patient was diagnosed with urinary tract infection (UTI) for the time being. Subsequent results showed normal values, so the diagnosis was withdrawn.

Another blood test showed a slightly higher creatinine level of 120 μM. The accepted range was 62–115 μM, an extremely high SGPT/ALT (serum glutamic-pyruvic transaminase/alanine aminotransferase) of 374 U/L compared to its normal range of 16–63 U/L. In contrast, the sodium level was slightly lower than acceptable, which was done to detect and diagnose liver abnormalities. After 3 days, subsequent testing showed normal labels of creatinine, HDL cholesterol, and blood urea nitrates. Nonetheless, the LDL cholesterol was high (4.2 mM compared to 0–3.3 mM), a hemoglobin A1c test showed 8.1%, and a high fasting blood sugar of 8.00 mM, diagnosing the Patient as diabetic, which was previously unknown.

The arterial blood gas testing was done twice, with all values ranging within healthy limits, except those of paO_2_, which was marked low at 67 (normal range: 80–100), oxygen saturation low at 91%, and a slightly lower HCO_3_ and paCO_2_. Oxygen saturation returned to normal in the subsequent test. However, HCO_3_ and paCO_2_ remained slightly low. Troponin and CK MB (Creatine kinase-MB) tests were also performed to detect and monitor heart attacks, or any damage to the heart, just as a precautionary step and the values were normal.

Although the Patient did not complain of any respiratory issues, a radiology test (X-ray) was performed following the positive COVID-19 test, and an ill-defined density at the lower left lobe characteristic of pneumonia was found. Hence, pneumonia was confirmed as a diagnosis and caused due to SARS-CoV-2, but the infection was only moderate.

## Discussion

The COVID-19 pandemic in dengue-endemic regions is cause for much concern, not only because of the difficulty in distinguishing between the two due to common clinical symptoms and laboratory characteristics but also because the risk of coinfection manifests severe disease symptoms and greater fatalities than single infections.

A coinfection case calls for more complex patient management and care needs in a country like the Philippines, where COVID-19 and dengue are rampant simultaneously. The case presented here shows a patient with coinfection diagnosed primarily as a COVID-19 patient, showing no major signs or symptoms of dengue and tested for that only on the Patient's insistence. The Patient had no major respiratory issues and was not hospitalized but sent home to recuperate in complete isolation, with regular telecommunication and follow-ups. This Patient's complete blood picture demonstrates no significant blood abnormalities but shows signs of thrombocytopenia, a typical condition in dengue infections, without which the physicians would not have assumed dengue infection. The extremely high levels of blood ALT were also indicative since liver injury is common in dengue due to a specific attack on the hepatic tissues by the DENV. Despite a lack of any history of chronic diseases, the tests also revealed the Patient to have diabetes at the time being, as shown by the high blood sugar, triglycerides, and hemoglobin A1c test. After 3 weeks, however, the tests showed contrasting results. The Patient's blood sugar returned to normal, confirmed by measurement on five consecutive days, suggesting that the momentary rise in blood sugar and triglycerides may be due to the infections. Still, it is hard to determine which virus or ^both^ were responsible for it. The poor oxygen saturation and low partial pressure of oxygen (PaO_2_) were indicative of the SARS-CoV-2 infection, a typical trait of COVID-19, and it returned to normal ranges within 3 weeks of diagnosis, causing no reason for concern. The progressive hematology reports show gradual and steady improvement of thrombocytopenia as platelet count increases. The physician gave a clearance report after 3 weeks as all vitals returned to normal, and the fever had also diminished, with body temperatures maintaining a steady value of 36.7°C for a few days. The Patient was still asked to remain in isolation for one more week, after which he got vaccinated for COVID-19.

The case presented here shows the unsuspected possibility of a COVID-19/dengue coinfection which could easily develop into a critical and morbid scenario. This Patient had a moderate to severe infection with no potentially fatal persistent symptoms and quickly recovered. Nonetheless, this portrays the possible challenges in discriminating COVID-19 from dengue because of their common, nonspecific nature of clinical symptoms. A similar case study of SARS-CoV-2–dengue virus coinfection was conducted in the Philippines (10). The 62 years old female in their report had a high fever, myalgia, and arthralgia and no rashes, cough, or dyspnoea, consistent with the Patient's symptoms in our study. However, the 62-year-old female was suspected of being infected with DENV based on her social and environmental history, but the Patient in our study was only tested for dengue at the Patient's request.

In contrast, most hospitals required patients admitted with dengue fever to undergo COVID-19 screening as part of their protocol (11). Patients with merely an acute fever and non-specific systemic symptoms were initially suspected of contracting COVID-19. The lack of specific symptoms made distinguishing between COVID-19 and other tropical illnesses challenging. Therefore, patients suspected of SARS-CoV-2 infection should also have social and environmental history investigated for the possibility of unsuspected coinfection. A similar study has been reported in the Philippines, reflecting how research on coinfections with SARS-CoV-2 and arboviruses is scarce, particularly in countries with a high dengue burden. Although most dengue-endemic regions (the tropics and sub-tropics) often lack the funds and/or resources to ensure a differential diagnosis of both infections through RT-PCR, as coinfection cases become more common, the authorities need to notice this and facilitate the allocation of funds accordingly to avoid a possible co-epidemic. It is imperative to bring forward more case reports to shed light on the importance of early detection of such coinfections so that proper measures can be implemented. Through this case report, the authors are trying to spread the message that in dengue-endemic regions, patients with COVID-19 RT-PCR negative tests should go for mandatory dengue tests, while dengue positive-tested patients should do a COVID-19 RT-PCR to be sure of coinfection cases.

More studies and case reports are needed to analyze the effects, specific diagnosis points, tests, and proper prevention and management measures for such scenarios to be designed.

## Data Availability Statement

The raw data supporting the conclusions of this article will be made available by the authors, without undue reservation.

## Ethics Statement

The studies involving human participants were reviewed and approved by the Institutional Ethical Committee for Experimentations on Human, Animal, Microbes, Environment and Living Natural Sources, School of Life Sciences, and School of Agriculture and Mineral Sciences, Shahjalal University of Science and Technology, Sylhet 3114, Bangladesh. The patients/participants provided their written informed consent to participate in this study. Written informed consent was obtained from the individual(s) for the publication of any potentially identifiable images or data included in this article.

## Author Contributions

YA conceived the study. CP, YA, and MH designed the study. CZ and MH supervised the study. CP, NA, and YA wrote the draft manuscript. NA, YA, ZY, CZ, JZ, and MH did the revisions. All authors approved the final version of the manuscript.

## Conflict of Interest

ZY was employed by Shanghai Omics Biotechnology Co., Ltd. The remaining authors declare that the research was conducted in the absence of any commercial or financial relationships that could be construed as a potential conflict of interest.

## Publisher's Note

All claims expressed in this article are solely those of the authors and do not necessarily represent those of their affiliated organizations, or those of the publisher, the editors and the reviewers. Any product that may be evaluated in this article, or claim that may be made by its manufacturer, is not guaranteed or endorsed by the publisher.

## References

[1] Sohrabi C Alsafi Z O'Neill N Khan M Kerwan A Al-Jabir A. World health organization declares global emergency: a review of the 2019 novel coronavirus (COVID-19). Int J Surg. (2020) 76:71–6. 10.1016/j.ijsu.2020.02.034 32112977 PMC7105032

[2] Masyeni S Santoso MS Widyaningsih PD Asmara DW Nainu F Harapan H. Serological cross-reaction and coinfection of dengue and COVID-19 in Asia: experience from Indonesia. Int J Infect Dis. (2021) 102:152–4. 10.1016/j.ijid.2020.10.043 33115680 PMC7585717

[3] Official WHO Coronavirus (COVID-19) Dashboard. Available online at: https://covid19.who.int/ (accessed February 3, 2022).

[4] Mushtaque RS Ahmad SM Mushtaque R Baloch S. A curious case of dengue fever: a case report of unorthodox manifestations. Case Rep Med. (2020) 2020:1701082. 10.1155/2020/1701082 32774384 PMC7399777

[5] World Health Organization. Global Strategy for Dengue Prevention and Control 2012–2020. WHO (2012).

[6] Tuiskunen Bäck A Lundkvist A. Dengue viruses – an overview. Infect Ecol Epidemiol. (2013) 3:19839. 10.3402/iee.v3i0.19839 24003364 PMC3759171

[7] Nasomsong W Luvira V Phiboonbanakit D. Case report: dengue and COVID-19 coinfection in Thailand. Am J Trop Med Hyg. (2021) 104:487–9. 10.4269/ajtmh.20-1340 33331264 PMC7866353

[8] Edillo FE Halasa YA Largo FM Erasmo JNV Amoin NB Alera MTP. Economic cost and burden of dengue in the Philippines. Am J Trop Med Hyg. (2015) 92:360–6. 10.4269/ajtmh.14-0139 25510723 PMC4347342

[9] Lustig Y Keler S Kolodny R Ben-Tal N Atias-Varon D Shlush E. Potential antigenic cross-reactivity between severe acute respiratory syndrome coronavirus 2 (SARS-CoV-2) and dengue viruses. Clin Infect Dis. (2021) 73:e2444–9. 10.1093/cid/ciaa1207 32797228 PMC7454334

[10] Saipen AL Demot B De Leon L. Dengue–COVID-19 coinfection: the first reported case in the Philippines. WPSAR. (2021) 12:35–9. 10.5365/wpsar.2020.11.3.016 34094622 PMC8143936

[11] Malibari AA Al-Husayni F Jabri A Al-Amri A Alharbi M. A patient with dengue fever and COVID-19: coinfection or not? Cureus. (2020) 12:e11955. 10.7759/cureus.11955 33312826 PMC7723426

